# Association between hematological inflammatory markers and latent TB infection: insights from NHANES 2011–2012 and transcriptomic data

**DOI:** 10.3389/fcimb.2025.1556048

**Published:** 2025-03-19

**Authors:** Yang Liu, Chunyan He, He Zhao, Weiyao Zhong, Shihua Sun, Zhuo Li, Jingwei Shi

**Affiliations:** ^1^ Department of Laboratory Medicine Center, China-Japan Union Hospital of Jilin University, Changchun, China; ^2^ Department of Clinical Laboratory, Kunshan Hospital of Chinese Medicine, Affiliated Hospital of Yangzhou University, Kunshan, Jiangsu, China

**Keywords:** latent tuberculosis infection, inflammation, NHANES, transcriptome, systemic immune-inflammation index

## Abstract

**Background:**

Latent tuberculosis infection affects about one-quarter of the global population and can progress to active tuberculosis. Hematological inflammatory markers, such as the systemic immune-inflammation index, neutrophil-to-lymphocyte ratio, platelet-to-lymphocyte ratio, and monocyte-to-lymphocyte ratio, reflect systemic inflammation and immune status but are understudied in latent tuberculosis infection. This study investigates the association between these markers and latent tuberculosis infection in a nationally representative sample.

**Methods:**

Data from 7,042 participants in the 2011–2012 National Health and Nutrition Examination Survey and transcriptomic data from the GSE19491 dataset were analyzed. Latent tuberculosis infection was identified using the QuantiFERON-TB Gold assay. Hematological parameters were measured via complete blood counts, and inflammatory markers were calculated through these parameters. Statistical analyses included linear regression adjusted for confounders and subgroup analyses. Transcriptomic analyses involved immune cell profiling, gene set enrichment, and immune checkpoint gene expression.

**Results:**

Individuals with latent tuberculosis infection had significantly lower systemic immune-inflammation index, neutrophil-to-lymphocyte ratio, platelet-to-lymphocyte ratio, and monocyte-to-lymphocyte ratio. These associations remained significant after adjusting for age, gender, body mass index, diabetes, and hypertension. Transcriptomic analyses revealed heightened activation of memory CD4 and CD8 T cells, increased cytolytic activity, and upregulated T-cell co-inhibition pathways, alongside differential expression of immune checkpoint genes in individuals with latent tuberculosis infection.

**Conclusions:**

A lower systemic immune-inflammation index and other related hematological inflammatory markers independently correlate with latent tuberculosis infection. These findings underscore the potential significance of hematological inflammatory markers in identifying and understanding latent tuberculosis infection. Further exploration of these markers may enhance diagnostic and therapeutic strategies of tuberculosis.

## Introduction

Latent tuberculosis infection (LTBI) represents a significant global health challenge and remains a key focus of efforts to eradicate tuberculosis (TB) (Hook, 2022). Approximately one-quarter of the world’s population is estimated to be infected with Mycobacterium tuberculosis in its latent form (Houben and Dodd, 2016). Individuals with LTBI are asymptomatic and non-infectious; however, they carry a 5–10% lifetime risk of progressing to active TB disease, especially within the first two years after infection (Wallgren, 1948; Dannenberg, 1994). This progression from LTBI to active TB significantly contributes to the global TB burden, which remains one of the leading causes of infectious disease-related mortality worldwide (Global tuberculosis report, 2023). Therefore, learning comprehensive insights into immune regulation and systemic inflammation in LTBI is essential for constructing effective therapies to prevent progressing to active TB and lessen transmission.

Emerging hematological ratios such as the systemic immune-inflammation index (SII), neutrophil-to-lymphocyte ratio (NLR), platelet-to-lymphocyte ratio (PLR), and monocyte-to-lymphocyte ratio (MLR) have been extensively studied for their role in assessing systemic inflammation and immune responses (Templeton et al., 2014; Fest et al., 2018; Tao et al., 2024). The systemic immune-inflammation index (SII), which integrates neutrophil, platelet, and lymphocyte counts into a single metric, has recently emerged as a novel indicator providing a more comprehensive assessment of the immune-inflammatory status (Hu et al., 2014; Chen et al., 2017; Xie et al., 2022). Due to their accessibility, cost-effectiveness, and rapid turnaround, SII, NLR, PLR, and MLR have become valuable tools in clinical practice for evaluating the prognosis of various conditions, including cancers, cardiovascular diseases, and infectious disorders.

The National Health and Nutrition Examination Survey (NHANES) database has been widely used in research on various diseases, particularly in analyzing cardiovascular diseases, diabetes, and respiratory diseases, with significant findings. For example, studies have demonstrated the important roles of blood inflammatory markers, such as the NLR, PLR, and SII, in heart failure and other chronic conditions (Ke et al., 2023; Xu et al., 2024). However, the application of these inflammatory markers in the study of LTBI remains limited. Existing research has primarily focused on the relationships between LTBI and factors like disease prevalence (Woodruff and Miramontes, 2023), smoking exposure (Hu et al., 2024), and hypertension (Salindri et al., 2024), with few studies systematically analyzing the association between these blood inflammatory markers and LTBI.

Therefore, this study seeks to fill this gap by examining these associations and comparing the predictive values of these biomarkers for LTBI. By analyzing data from the NHANES database and the GSE19491 dataset, we aim to enhance the understanding of how immune-inflammatory markers relate to LTBI in a nationally representative sample of the U.S. population.

## Materials and methods

### Study design and population

**Figure 1:**
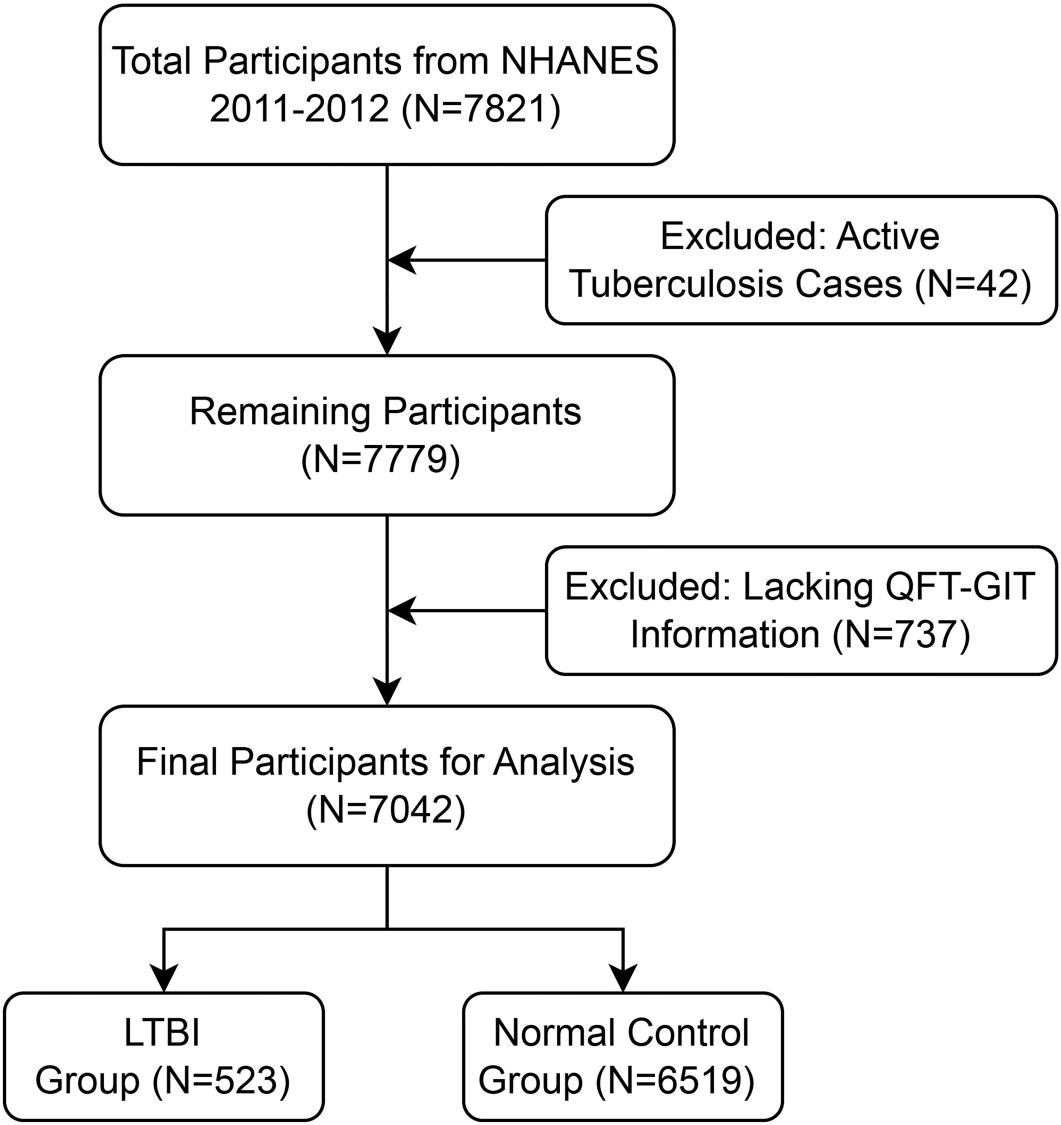
Flowchart of participant selection for the study.

This study utilized data from the 2011–2012 National Health and NHANES, a thorough, widely representative survey aimed at assessing the health, nutritional, and demographic characteristics in the U.S. population (NHANES 2011-2012 overview). NHANES uses a complex, multistage probability sampling method to ensure comprehensive representation, with data collected via structured interviews, anonymous surveys, mobile examination centers (MEC) doing physical evaluations, and extensive laboratory analyses. Full documentation about the NHANES framework and methods is accessible online (www.cdc.gov/nchs/nhanes/). A total of 7,821 participants were initially included in the analysis. Subsequently, the sample size was reduced to 7,779 after excluding 42 individuals diagnosed with active TB. Further exclusion of 737 respondents due to incomplete data on LTBI resulted in a final cohort of 7,042 individuals. Among these, 523 participants were identified with LTBI, while 6,519 served as the control group without LTBI. The selection process is depicted in the flowchart (**Figure 1**).

The GEO dataset was downloaded from the GEO database (http://www.ncbi.nlm.nih.gov/geo). The GSE19491 dataset contains blood transcriptional profiles from individuals with LTBI and healthy controls. And it includes 69 samples from LTBI patients and 36 samples from healthy controls (HC) without LTBI.

### Definition of LTBI

The QuantiFERON-TB Gold In-Tube (QFT-GIT) assay was performed following the manufacturer’s instructions. Blood samples were incubated with TB antigens at 37°C for 16–24 hours, and interferon-gamma (IFN-γ) levels were measured using ELISA. Result interpretation adhered to Centers for Disease Control and Prevention (CDC) guidelines for interferon-gamma release assays (IGRAs). Participants with positive QFT-GIT results were classified as LTBI positive, while those with negative results were deemed LTBI negative. For indeterminate or missing QFT-GIT results, individuals were considered LTBI positive if their purified protein derivative (PPD) test showed an induration of at least 10 mm, regardless of LTBI risk factors (Khan et al., 2008; Zheng et al., 2020).

### Peripheral blood cell counts and immune-inflammatory markers

In accordance with the protocol of the study, white blood cell (WBC), neutrophil (NEU), lymphocyte (LYM), monocyte (MON), and platelet (PLT) counts were measured using complete blood count (CBC) assays performed on automated hematology analyzers, such as the Coulter DxH 800 analyzer. Blood specimens were collected and processed following the guidelines of the National Health and NHANES at Mobile Examination Centers (MECs). The counts are presented as ×109/L\times 10^9/L×109/L. Additionally, the percentages of lymphocytes, neutrophils, and monocytes were recorded.

### Covariates

Covariates included age, gender, race, education level, income-to-poverty ratio, Body Mass Index (BMI), waist circumference, smoking status (defined as having smoked at least 100 cigarettes), diabetes status, and hypertension status.

### Transcriptome data analysis

To further understand the proportions of various immune cell types in the blood samples, we utilized the R package CIBERSORT (Newman et al., 2015) along with the Leukocyte Signature Matrix (LM22) (Chen et al., 2018), which comprises 547 reference genes. This approach allowed us to calculate the abundance of different immune cells in each sample, thereby gaining insights into the immune cell composition associated with LTBI.

To investigate immune-related functional differences between LTBI and healthy control samples, we employed single-sample Gene Set Enrichment Analysis (ssGSEA) (Hänzelmann et al., 2013). This method enabled us to assess the enrichment of various immune cell types and functions in each group, providing a deeper understanding of the immune responses involved in LTBI.

Furthermore, we analyzed the expression differences of immune checkpoint genes between LTBI samples and healthy controls to explore potential immunoregulatory mechanisms contributing to LTBI persistence or protection. A total of 47 immune checkpoint genes were selected for this analysis, including key regulators such as CD44, TMIGD2 and TNFSF14 (Hu et al., 2021).

### Statistical analysis

All statistical analyses were conducted using R software (version 4.3.0). Continuous variables were expressed as mean ± standard deviation (SD) for normally distributed data or as median with interquartile range (IQR) for non-normally distributed data. Categorical variables were presented as frequencies and percentages.

Based on these peripheral blood cell counts, we calculated four immune-inflammatory markers: SII, NLR, PLR, and MLR. The calculations were as follows: SII = (neutrophils × platelets)/lymphocytes, NLR = neutrophils/lymphocytes, PLR = platelets/lymphocytes, and MLR = monocytes/lymphocytes. The multivariate test was created using three models: model 1: no variables adjusted; model 2: gender, age, and race adjusted; and model 3: adjusted for all covariates.

Subgroup analyses were conducted to evaluate potential disparities across different populations. Specifically, we examined the associations between SII, lymphocyte percentage, PLR, MLR, and NLR with LTBI in relation to age group, gender, body mass index (BMI) category, diabetes status, and hypertension status. Interaction tests were performed to determine whether these variables significantly influenced these associations.

## Results

### Demographic and clinical characteristics

In our study, 7,042 adults were included based on the inclusion and exclusion criteria, with a median age of 35.0 years (IQR: 17.0–56.0 years). Among these participants, 49.8% were men and 50.2% were women. The median (IQR) systemic immune-inflammation index (SII) was 420 × 10^9/L (IQR: 295–612). The median (IQR) values of SII, NLR, PLR, and MLR were 420 (295–612), 1.79 (1.29–2.47), 116 (93.0–146), and 0.24 (0.18–0.30), respectively.

### Lower SII, NLR, PLR, MLR levels and higher lymphocyte percentages associated with latent tuberculosis infection

**Table 1** details all study population characteristics based on latent tuberculosis infection status. In comparison to the healthy control group, the LTBI group is more likely to be male and older; with higher proportions of Other Race—including Multi-Racial and Other Hispanic ethnicity; with a lower educational level; higher smoking status; higher levels of BMI and waist circumference; higher prevalence of diabetes and hypertension; and lower levels of SII, PLR, WBCs, platelets, and lymphocytes.

**Table 1:** Demographic and clinical characteristics of the study population stratified by LTBI status.

	HC	LTBI	p.overall
	N=6519	N=523	
SII (10^9/L)	421 [296;615]	410 [288;564]	0.038
NLR	1.78 [1.29;2.47]	1.81 [1.33;2.44]	0.352
PLR	117 [93.1;146]	110 [91.8;135]	0.003
MLR	0.24 [0.18;0.30]	0.24 [0.18;0.30]	0.562
WBCs (10^9/L)	6.60 [5.50;8.10]	6.40 [5.40;7.80]	0.046
Neutrophils (10^9/L)	3.70 [2.80;4.80]	3.60 [2.80;4.77]	0.591
PLT (10^9/L)	240 [205;282]	222 [189;260]	<0.001
Lymphocytes (10^9/L)	2.10 [1.70;2.60]	2.00 [1.60;2.40]	0.015
Monocytes (10^9/L)	0.50 [0.40;0.60]	0.50 [0.40;0.60]	0.167
Lymphocyte_Percentage	31.8 [25.7;38.5]	31.5 [25.9;37.7]	0.383
Neutrophil_Percentage	56.7 [49.3;63.8]	57.0 [50.2;63.5]	0.357
Monocyte Percentage	7.50 [6.10;8.90]	7.50 [6.20;8.80]	0.748
Gender *(%)*			<0.001
Female	3312 (50.8%)	221 (42.3%)	
Male	3207 (49.2%)	302 (57.7%)	
Age (years)	33.0 [16.0;54.0]	57.0 [42.0;67.0]	<0.001
Race/Ethnicity * (%)*			<0.001
Mexican American	814 (12.5%)	80 (15.3%)	
Non-Hispanic Black	1783 (27.4%)	123 (23.5%)	
Non-Hispanic White	2255 (34.6%)	76 (14.5%)	
Other Hispanic	646 (9.91%)	93 (17.8%)	
Other Race - Including Multi-Racial	1021 (15.7%)	151 (28.9%)	
Education level			<0.001
< High school	945 (21.3%)	184 (37.2%)	
> High school	3483 (78.7%)	311 (62.8%)	
Income to poverty ratio	1.74 [0.92; 3.78]	1.65 [0.89; 3.51]	0.218
BMI (kg/m2)	25.7 [21.3; 30.6]	27.0 [23.9; 31.7]	<0.001
Waist circumference (cm)	90.1 [76.4;104]	96.0 [86.6;106]	<0.001
Smoked at least 100 cigarettes:			0.038
No	2556 (57.7%)	261 (52.7%)	
Yes	1874 (42.3%)	234 (47.3%)	
Diabetes:			<0.001
No	5985 (91.8%)	430 (82.2%)	
Yes	534 (8.19%)	93 (17.8%)	
Hypertension:			<0.001
No	3383 (68.2%)	301 (59.4%)	
Yes	1577 (31.8%)	206 (40.6%)	

HC, Healthy Control; LTBI, Latent Tuberculosis Infection; SII, Systemic Immune-Inflammation Index; NLR, Neutrophil to Lymphocyte Ratio; PLR, Platelet to Lymphocyte Ratio; MLR, Monocyte to Lymphocyte Ratio; WBCs, White Blood Cells; PLT, Platelets; BMI, Body Mass Index.

In this study, the unadjusted (Model 1) linear association analysis demonstrated a significant negative correlation between SII and LTBI status, yielding a β coefficient of -34.46 (95% CI: -63.47, -5.45; P = 0.020). This association was consistently observed in Models 2 and 3. In the fully adjusted model (Model 3), which accounted for all confounders, the association between SII and LTBI status remained significant, with a β coefficient of -44.91 (95% CI: -80.17, -9.65; P = 0.013). This suggests that individuals with LTBI have significantly lower SII levels compared to healthy controls (**Table 2**).

**Table 2:** Associations between immune-inflammatory markers and latent tuberculosis infection (LTBI) status in linear regression models.

Variable	Model 1 β (95% CI) P value	Model 2 β (95% CI) P value	Model 3 β (95% CI) P value
SII (10^9/L)	-34.46 (-63.47, -5.45) 0.020	-62.40 (-91.93, -32.86) <0.001	-44.91 (-80.17, -9.65) 0.013
NLR	-0.03 (-0.14, 0.08) 0.582	-0.25 (-0.35, -0.14) <0.001	-0.18 (-0.30, -0.05) 0.008
PLR	-5.94 (-10.20, -1.67) 0.006	-8.53 (-12.92, -4.13) <0.001	-6.03 (-11.23, -0.82) 0.023
MLR	-0.003 (-0.014, 0.008) 0.566	-0.020 (-0.031, -0.009) <0.001	-0.019 (-0.031, -0.007) 0.002
WBCs (10^9/L)	-0.21 (-0.41, -0.02) 0.035	-0.19 (-0.39, 0.01) 0.059	-0.19 (-0.42, 0.04) 0.106
Neutrophils (10^9/L)	-0.09 (-0.25, 0.06) 0.216	-0.21 (-0.36, -0.06) 0.007	-0.15 (-0.32, 0.02) 0.083
PLT (10^9/L)	-17.46 (-23.03, -11.89) 0.000	-4.08 (-9.60, 1.43) 0.147	-2.92 (-9.09, 3.26) 0.354
Lymphocytes (10^9/L)	-0.09 (-0.18, -0.004) 0.040	0.03 (-0.06, 0.12) 0.561	-0.01 (-0.13, 0.10) 0.821
Monocytes (10^9/L)	-0.02 (-0.04, 0.005) 0.131	-0.01 (-0.03, 0.008) 0.242	-0.02 (-0.05, 0.003) 0.081
Lymphocyte Percentage (%)	-0.47 (-1.32, 0.37) 0.274	1.56 (0.74, 2.37) <0.001	1.06 (0.17, 1.95) 0.019
Neutrophil Percentage (%)	0.56 (-0.40, 1.53) 0.251	-1.62 (-2.56, -0.69) 0.001	-0.89 (-1.90, 0.13) 0.087
Monocyte Percentage (%)	0.01 (-0.22, 0.23) 0.955	0.07 (-0.16, 0.29) 0.573	-0.09 (-0.34, 0.17) 0.500

Model 1 is unadjusted; Model 2 is adjusted for age, gender, and race, and; Model 3 is further adjusted for education level, family income-to-poverty ratio, body mass index (BMI), smoking status, waist circumference, diabetes, and hypertension.

Similarly, NLR showed a significant negative association with LTBI status in the adjusted models. In Model 2, the β coefficient was -0.25 (95% CI: -0.35, -0.14; P < 0.001), and in Model 3, it was -0.18 (95% CI: -0.30, -0.05; P = 0.008). Furthermore, MLR exhibited a non-significant association in Model 1 but showed significant negative correlations in Models 2 and 3, with β coefficients of -0.020 (P < 0.001) and -0.019 (P = 0.002) respectively, suggesting a decrease in MLR with LTBI status. In addition, PLR also demonstrated a significant negative association across all models. In Model 1, the β coefficient was -5.94 (95% CI: -10.20, -1.67; P = 0.006), which remained significant in Models 2 and 3.

Additionally, lymphocyte percentage exhibited a significant positive association with LTBI status in the adjusted models. The β coefficient was 1.56 (95% CI: 0.74, 2.37; P < 0.001) in Model 2 and 1.06 (95% CI: 0.17, 1.95; P = 0.019) in Model 3, indicating there is a positive association between lymphocyte percentage and LTBI status.

In conclusion, these findings indicate that LTBI status is associated with lower levels of SII, NLR, MLR and PLR (**Table 2**).

### Subgroup analysis

Specific conditions were evaluated to address potential disparities across these populations, and we examined relationships with age group, gender, body mass index (BMI) category, diabetes status, and hypertension status. The correlations between SII and LTBI remained consistent across subgroups (**Table 3**). These associations were not significantly affected by age group, gender, BMI group, diabetes status, or hypertension status (P for interaction > 0.05). Subgroup analyses for the PLR, MLR, and NLR delivered a similar result (**Supplementary Tables S1–S3**).

**Table 3:** Subgroup analysis of the association between systemic immune-inflammation index (SII) and LTBI status across different populations.

Variable	β (95% CI)	P value	P for interaction
Age Group (years)			0.078
<60	-24.85 (-64.77, 15.07)	0.222	
>=60	-73.43 (-141.6, -5.27)	0.035	
Gender			0.244
Female	-59.9 (-116.73, -3.07)	0.039	
Male	-21.06 (-63.36, 21.23)	0.329	
BMI Group (kg/m2)			0.483
<25	-96.36 (-283.71, 90.99)	0.314	
>=25	-40.64 (-76.51, -4.77)	0.026	
Diabetes			0.162
No	-33.55 (-71.33, 4.23)	0.082	
Yes	-99.72 (-194.02, -5.42)	0.039	
Hypertension			0.134
No	-27.22 (-66.63, 12.19)	0.176	
Yes	-70.01 (-135.91, -4.1)	0.038	

The association between SII and LTBI status was examined within subgroups defined by age, gender, BMI, diabetes status, and hypertension status. Interaction P-values assess whether the association differs significantly across subgroups.

### Immune cell profiles in blood based on transcriptomics

**Figure 2:**
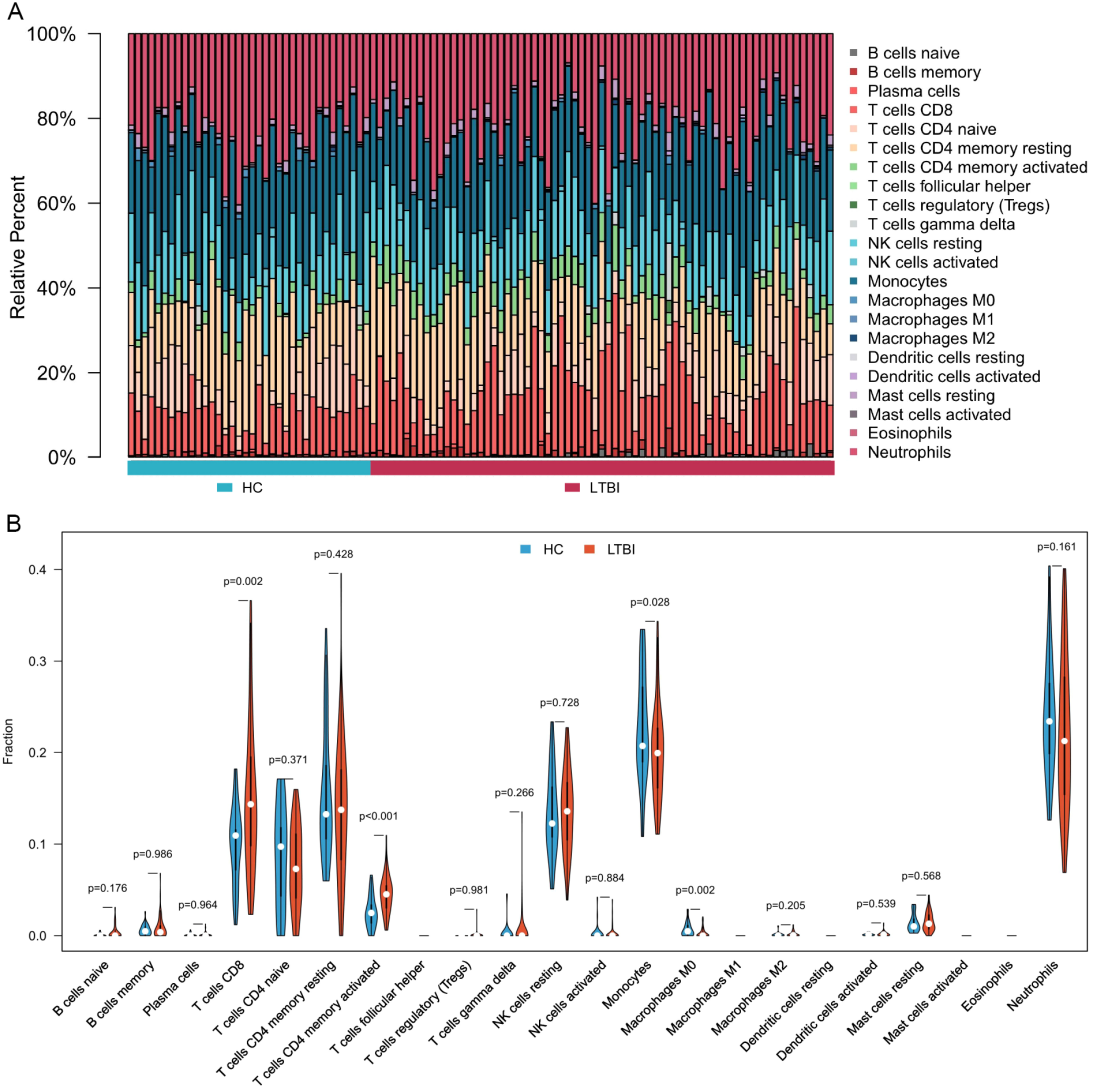
Immune cell composition and differences between LTBI samples and healthy controls. **(A)** Bar chart illustrating the composition of immune cells in each sample. **(B)** Comparative analysis of immune cell proportions between LTBI samples and healthy controls.

**Figure 2A** displays the abundance of various blood immune cell categories in each sample, determined by the CIBERSORT algorithm. In comparison with healthy controls, LTBI samples exhibited a significantly higher proportion of activated memory CD4^+^ T cells and CD8^+^ T cells. Conversely, the proportion of monocytes was slightly reduced in LTBI samples relative to healthy controls (P < 0.05) (**Figure 2B**).

### Immune function analysis

**Figure 3:**
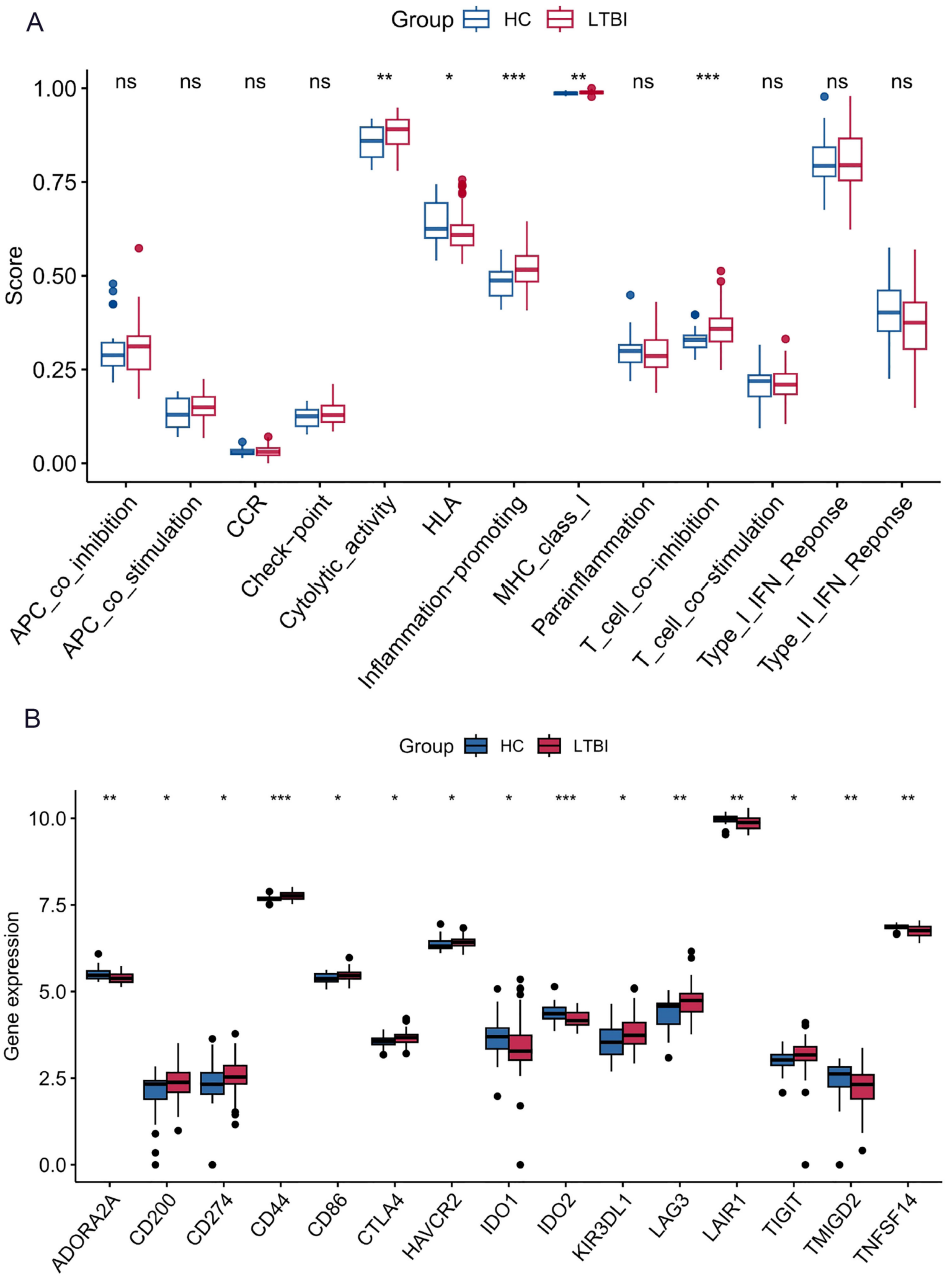
Immune function differences and immune checkpoint gene expression between LTBI samples and healthy controls. **(A)** Immune function analysis showing differences between LTBI samples and healthy controls. **(B)** Differential expression analysis of immune checkpoint genes between LTBI samples and healthy controls. *p < 0.05, **p < 0.01, ***p < 0.001.

**Figure 3A** indicates that immune functions, including cytolytic activity (p < 0.01), inflammation-promoting (p < 0.001), and T cell co-inhibition (p < 0.001), were significantly elevated in LTBI samples compared to healthy controls. Conversely, human leukocyte antigen (HLA) expression was significantly lower in LTBI samples than in the healthy group (p < 0.05).

### Analysis of immune checkpoint differences

The results showed that several immune checkpoint genes, including CD44, TIGIT, and LAG3, were highly expressed in LTBI samples compared to healthy controls. In contrast, genes such as IDO2, ADORA2A, LAIR1, and TNFSF14 exhibited lower expression levels in LTBI samples than in the healthy group (**Figure 3B**).

## Discussion

Tuberculosis remains a major global health challenge, as numerous individuals with LTBI may progress to active disease (Getahun et al., 2015; Fox et al., 2017). Hematological parameters derived from routine blood tests, such as SII, NLR, PLR, and MLR, are widely recognized for their ability to reflect systemic inflammation and immune status. However, their specific associations with LTBI have not been extensively studied until now.

Analysis of NHANES 2011–2012 and GSE19491 data revealed that individuals with LTBI have distinct immune-inflammatory profiles compared to those without LTBI. Utilizing the nationally representative sample of U.S. adults, our study discovered that individuals with LTBI exhibit significantly lower levels of SII, NLR, PLR, and MLR compared to those without LTBI. Specifically, fully adjusted linear models revealed inverse associations between LTBI status and SII (β = −44.91; 95% CI: −80.17 to −9.65; P = 0.013), NLR (β = −0.18; 95% CI: −0.30 to −0.05; P = 0.008), PLR, and MLR. These findings suggest that LTBI is characterized by a lower systemic inflammatory state.

In the clinical assessment of infectious diseases, hematological markers such as SII (Liu et al., 2023; Yang et al., 2024), NLR (Buonacera et al., 2022; Chen et al., 2023), PLR (Russell et al., 2019; Sarkar et al., 2022) and MLR (Mao and Wu, 2020; Li et al., 2024) typically rise in response to increased disease severity and are prognostic of poorer outcomes. In latent tuberculosis infection (LTBI), these markers are notably reduced, suggesting that LTBI represents a controlled and contained immune response that effectively manages the infection without triggering severe inflammation.

To further explore the specific hematological immune characteristics of LTBI, we conducted transcriptomic analyses, which provided additional evidence supporting our findings. These analyses revealed that individuals with LTBI exhibit increased proportions of activated memory CD4^+^ and CD8^+^ T cells, alongside enhanced immune functions such as cytolytic activity and T-cell co-inhibition. This aligns with previous studies demonstrating that enhanced cytolytic activity and regulatory mechanisms play a critical role in maintaining immune homeostasis in chronic infections (Bolivar-Wagers et al., 2022; Teh et al., 2022). Moreover, we identified differential expression of immune checkpoint genes, including upregulation of CD44, TIGIT, and LAG3, and downregulation of IDO2, ADORA2A, LAIR1, and TNFSF14. These molecular changes suggest an immune environment optimized for pathogen control without excessive inflammation. The role of CD44 in modulating immune responses has been highlighted in various settings, including its involvement in macrophage polarization and immune cell infiltration, which supports its relevance in LTBI (Leemans et al., 2003). Additionally, LAG3, as a critical immune checkpoint, is known to regulate T-cell activation and maintain a balanced immune response, preventing immune exhaustion in chronic condition (Ruffo et al., 2019). These molecular alterations likely create an immune environment that effectively controls bacterial proliferation while avoiding the development of severe inflammation.

The cross-sectional design of the NHANES data limits our capacity for determining causation between immune-inflammatory indicators and LTBI. Therefore, longitudinal studies are needed in the future to further investigate and confirm the causal relationship between these two factors. While we adjusted for numerous potential confounders, unmeasured variables may still influence the associations observed. The diagnosis of LTBI was based on the QFT-GIT assay, which despite its widespread use has limitations in sensitivity and specificity. Additionally, we lacked information on the duration of infection and prior exposure history, which could affect immune responses.

Despite these constraints, this research offers important insights into the immune-inflammatory dynamics of LTBI in a large, nationally representative cohort. The integration of transcriptomic data adds a molecular dimension to our understanding, revealing specific immune cell alterations and functional changes associated with LTBI. The application of new technologies will further improve our capacity to explore immune features in detail (Liu et al., 2023). These findings have significant implications for public health strategies aimed at tuberculosis control. The identified hematologic markers, being readily available and cost-effective, could serve as supplementary tools for LTBI screening and risk assessment, particularly in resource-limited settings. They could help identify individuals who require additional monitoring or early treatment to prevent the development of active tuberculosis.

## Conclusion

In conclusion, our findings demonstrate that lower levels of SII, NLR, PLR, and MLR are independently associated with LTBI. These hematologic inflammatory markers reflect a state of immune equilibrium that effectively contains the latent infection. Additionally, our transcriptomic analyses revealed increased activation of memory CD4^+^ and CD8^+^ T cells, enhanced immune functions such as cytolytic activity in LTBI individuals, further emphasizing the active role of the immune system in maintaining latency. Future prospective studies are warranted to validate these associations and explore their potential in guiding interventions aimed at preventing the progression to active tuberculosis.

## Data Availability

The datasets presented in this study can be found in online repositories. The NHANES 2011-2012 dataset can be accessed via the link: https://wwwn.cdc.gov/nchs/nhanes/. The GEO Series accession number GSE19491 is available at https://www.ncbi.nlm.nih.gov/geo/.

## Supplementary material

Supplementary Table S1–3 Subgroup Analyses of the Associations Between Platelet-to-Lymphocyte Ratio (PLR), Monocyte-to-Lymphocyte Ratio (MLR), Neutrophil-to-Lymphocyte Ratio (NLR), and LTBI Status Across Different Populations. These tables present the results of subgroup analyses examining the associations between PLR (**Supplementary Table S1**), MLR (**Supplementary Table S2**), and NLR (**Supplementary Table S3**) with LTBI status within subgroups defined by age, gender, body mass index (BMI), diabetes status (yes, no), and hypertension status. Interaction P-values assess whether these associations differ significantly across subgroups.
