# Supplementary material for: Association between hematological inflammatory markers and latent TB infection: insights from NHANES 2011–2012 and transcriptomic data
Source: Front Cell Infect Microbiol. 2025 Mar 19;15:1556048. doi: 10.3389/fcimb.2025.1556048 (PMC11962010; doi:10.3389/fcimb.2025.1556048)
Supplement: Supplementary Table S1–3 — Subgroup Analyses of the Associations Between Platelet-to-Lymphocyte Ratio (PLR), Monocyte-to-Lymphocyte Ratio (MLR), Neutrophil-to-Lymphocyte Ratio (NLR), and LTBI Status Across Different Populations. These tables present the results of subgroup analyses examining the associations between PLR ( Supplementary Table S1 ), MLR ( Supplementary Table S2 ), and NLR ( Supplementary Table S3 ) with LTBI status within subgroups defined by age, gender, body mass index (BMI), diabetes status (yes, no), and hypertension status. Interaction P-values assess whether these associations differ significantly across subgroups. [file Table1.docx]

**Table S1**

| **Variable** | **Count** | **Percent** | **β (95% CI)** | **P value** | **P for interaction** |
| --- | --- | --- | --- | --- | --- |
| Age Group (years) | |  |  |  | 0.164 |
| <60 | 5498 | 78.10% | -2.87 (-9.09, 3.36) | 0.367 |  |
| >=60 | 1544 | 21.90% | -8.44 (-17.95, 1.08) | 0.083 |  |
| Gender |  |  |  |  | 0.147 |
| Female | 3533 | 50.20% | -7.42 (-15.50, 0.67) | 0.072 |  |
| Male | 3509 | 49.80% | 0.59 (-6.06, 7.25) | 0.861 |  |
| BMI Group (kg/m^2) | | |  |  | 0.281 |
| <25 | 2599 | 36.90% | -18.55 (-45.44, 8.34) | 0.177 |  |
| >=25 | 4443 | 63.10% | -3.44 (-8.79, 1.90) | 0.207 |  |
| Diabetes |  |  |  |  | 0.426 |
| No | 6415 | 91.10% | -2.77 (-8.48, 2.94) | 0.341 |  |
| Yes | 627 | 8.90% | -8.39 (-21.17, 4.38) | 0.199 |  |
| Hypertension | |  |  |  | 0.041 |
| No | 3684 | 67.40% | 0.04 (-6.13, 6.20) | 0.991 |  |
| Yes | 1783 | 32.60% | -9.06 (-18.30, 0.18) | 0.055 |  |

**Table S2**

| **Variable** | **Count** | **Percent** | **β (95% CI)** | **P value** | **P for interaction** |
| --- | --- | --- | --- | --- | --- |
| Age Group (years) | |  |  |  | 0.101 |
| <60 | 5498 | 78.10% | -0.01 (-0.02, 0.01) | 0.336 |  |
| >=60 | 1544 | 21.90% | -0.02 (-0.04, 0.00) | 0.073 |  |
| Gender |  |  |  |  | 0.226 |
| Female | 3533 | 50.20% | -0.02 (-0.03, 0.00) | 0.055 |  |
| Male | 3509 | 49.80% | 0.00 (-0.02, 0.02) | 0.964 |  |
| BMI Group (kg/m^2) | | |  |  | 0.416 |
| <25 | 2599 | 36.90% | 0.03 (-0.03, 0.08) | 0.398 |  |
| >=25 | 4443 | 63.10% | 0.00 (-0.02, 0.01) | 0.483 |  |
| Diabetes |  |  |  |  | 0.177 |
| No | 6415 | 91.10% | 0.00 (-0.02, 0.01) | 0.664 |  |
| Yes | 627 | 8.90% | -0.02 (-0.06, 0.01) | 0.226 |  |
| Hypertension | |  |  |  | 0.717 |
| No | 3684 | 67.40% | -0.01 (-0.02, 0.01) | 0.327 |  |
| Yes | 1783 | 32.60% | -0.01 (-0.03, 0.02) | 0.663 |  |

**Table S3**

| **Variable** | **Count** | **Percent** | **β (95% CI)** | **P value** | **P for interaction** |
| --- | --- | --- | --- | --- | --- |
| Age Group (years) | |  |  |  | 0.166 |
| <60 | 5498 | 78.10% | -0.1 (-0.24, 0.05) | 0.185 |  |
| >=60 | 1544 | 21.90% | -0.22 (-0.48, 0.04) | 0.091 |  |
| Gender |  |  |  |  | 0.251 |
| Female | 3533 | 50.20% | -0.18 (-0.38, 0.02) | 0.074 |  |
| Male | 3509 | 49.80% | -0.04 (-0.2, 0.13) | 0.677 |  |
| BMI Group (kg/m^2) | | |  |  | 0.779 |
| <25 | 2599 | 36.90% | -0.16 (-0.84, 0.52) | 0.65 |  |
| >=25 | 4443 | 63.10% | -0.1 (-0.23, 0.03) | 0.136 |  |
| Diabetes |  |  |  |  | 0.059 |
| No | 6415 | 91.10% | -0.07 (-0.2, 0.07) | 0.327 |  |
| Yes | 627 | 8.90% | -0.37 (-0.77, 0.03) | 0.069 |  |
| Hypertension | |  |  |  | 0.301 |
| No | 3684 | 67.40% | -0.08 (-0.22, 0.06) | 0.275 |  |
| Yes | 1783 | 32.60% | -0.16 (-0.41, 0.08) | 0.192 |  |
